# Antimicrobial Stewardship and Its Impact on the Changing Epidemiology of Polymyxin Use in a South Indian Healthcare Setting

**DOI:** 10.3390/antibiotics10050470

**Published:** 2021-04-21

**Authors:** Dipu T. Sathyapalan, Jini James, Sangita Sudhir, Vrinda Nampoothiri, Praveena N. Bhaskaran, Nandita Shashindran, Jisha Thomas, Preetha Prasanna, Zubair Umer Mohamed, Fabia Edathadathil, Sanjeev Singh, Merlin Moni

**Affiliations:** 1 Department of Medicine, Division of Infectious Diseases, Amrita Institute of Medical Science, Amrita Vishwa Vidyapeetham, Kochi 682041, Kerala, India; diputs19847@aims.amrita.edu (D.T.S.); preethap@aims.amrita.edu (P.P.); 2 Department of Infection Control and Epidemiology, Amrita Institute of Medical Science, Amrita Vishwa Vidyapeetham, Kochi 682041, Kerala, India; jinijames@aims.amrita.edu (J.J.); sangitas@aims.amrita.edu (S.S.); vrindan@aims.amrita.edu (V.N.); jishathomas@aims.amrita.edu (J.T.); fabiaet22441@aims.amrita.edu (F.E.); sanjeevksingh@aims.amrita.edu (S.S.); 3 Department of Pediatrics, Amrita Institute of Medical Sciences, Amrita Vishwa Vidyapeetham, Kochi 682041, Kerala, India; praveenanb@aims.amrita.edu; 4 Department of Microbiology, Amrita Institute of Medical Sciences, Amrita Vishwa Vidyapeetham, Kochi 682041, Kerala, India; nanditas@aims.amrita.edu; 5 Department of Anaesthesia and Critical Care, Amrita Institute of Medical Science, Amrita Vishwa Vidyapeetham, Kochi 682041, Kerala, India; zubairum22623@aims.amrita.edu

**Keywords:** polymyxins, antimicrobial stewardship, reserved drugs

## Abstract

Polymyxins being last resort drugs to treat infections triggered by multidrug-resistant pathogens necessitates the implementation of antimicrobial stewardship program (ASP) initiatives to support its rational prescription across healthcare settings. Our study aims to describe the change in the epidemiology of polymyxins and patient outcomes following the implementation of ASP at our institution. The antimicrobial stewardship program initiated in February 2016 at our 1300 bed tertiary care center involved post-prescriptive audits tracking polymyxin consumption and evaluating prescription appropriateness in terms of the right indication, right frequency, right drug, right duration of therapy and administration of the right loading dose (LD) and maintenance dose (MD). Among the 2442 polymyxin prescriptions tracked over the entire study period ranging from February 2016 to January 2020, the number of prescriptions dropped from 772 prescriptions in the pre-implementation period to an average of 417 per year during the post-implementation period, recording a 45% reduction. The quarterly patient survival rates had a significant positive correlation with the quarterly prescription appropriateness rates (r = 0.4774, *p* = 0.02), right loading dose (r = 0.5228, *p* = 0.015) and right duration (r = 0.4361, *p* = 0.04). Our study on the epidemiology of polymyxin use demonstrated favorable effects on the appropriateness of prescriptions and mortality benefits after successful implementation of antimicrobial stewardship in a real-world setting.

## 1. Introduction

The prevalence of healthcare-associated infections (HAIs) caused by antibiotic-resistant, gram-negative organisms, including extended spectrum β-lactamase-producing and carbapenem-resistant *Enterobacterales*, multidrug-resistant (MDR) *Acinetobacter baumannii* and *Pseudomonas aeruginosa*, is increasing worldwide and represents a major public health threat [1,2,3]. The global escalation of antimicrobial resistance (AMR) is unmatched in its pace with the discovery of equally effective antibiotics. The brunt of MDR gram-negative infections is high in the Indian setting, which could be attributed to widespread inappropriate antimicrobial prescriptions used in the treatment of gram-negative infections [4,5]. A vicious cycle is triggered in response to the treatment of MDR organisms, demanding the increased consumption of antimicrobials [6]. Therapeutic drug options for carbapenem-resistant infections are extremely limited and presently encompass the polymyxins (polymyxin B and colistin), which are now considered as last resort drugs under the WHO AWaRe Index [7,8] which classifies drugs based on antimicrobial resistance potential. Polymyxins are reported to be widely used in the treatment of difficult-to-treat (DTR) blood stream pathogens, implying no active first line agents and higher resistance relative to non-DTR isolates [9]. However, widespread inappropriate prescriptions in usual drug resistance (UDR) and non-standardized dosing regimens will also foster a risk of resistance. Optimum utilization of such antimicrobials that are possibly active against multidrug and extremely drug resistant (MDR and XDR) bacteria is consequently warranted. The Indian Council of Medical Research has identified carbapenems and polymyxins, namely polymyxin E and B (PE and PB), as key antimicrobials which require restriction in hospitals [10].

Despite international guidelines for the appropriate dosage of polymyxins, robust data regarding the optimal usage of these drugs from Indian settings are limited, which is further accentuated by poor stewardship practices [11,12,13]. Moreover, polymyxin dosing is a relatively arduous process, involving the follow-up of daily creatinine clearance rates with dose adjustments and close monitoring of patients with deranged renal parameters, encompassing acute kidney injury (AKI) and chronic kidney disease (CKD). For instituting appropriate colistin therapy in hemodialysis patients, continuous renal replacement therapy (CRRT) and sustained low-efficiency dialysis (SLED) to ensure appropriate steady state concentrations has always been a challenge due to lack of knowledge of guidelines among prescribers. Standardization of polymyxin prescriptions among treating physicians in the absence of dosage data is a major hurdle. Providing appropriate therapy is critical in patients with severe infections caused by MDR bacteria. Underdosing runs the risk of treatment failure, poor outcomes and the potential development and spread of antimicrobial resistance, including against polymyxins [14]. Considering that very few novel antibiotics covering MDR gram-negative bacteria can be expected to reach the market in the near future, it is essential to use the polymyxin class of antibiotics optimally and rationally [15]. Among various recommended strategies and nationwide policies adopted to combat AMR, the institution of stewardship practices has been recognized as an effective measure to ensure the appropriate use of antimicrobials in all healthcare setting levels, and epidemiological data on polymyxin consumption would be indicative of the sustained effectiveness of such strategies [16,17].

The antimicrobial stewardship program (ASP) established at our tertiary care institution in February 2016 is a multidisciplinary model driven by clinical pharmacists who monitor the appropriateness of the use of reserve antibiotics [18]. Institutional dosing guidelines for colistin and polymyxin B were compiled, implemented and disseminated through the hospital intranet for reference (Table S1).

We will describe the change in epidemiology of polymyxin B (PB) and polymyxin E (PE, or colistin) use and the patient outcomes following the implementation of the ASP.

## 2. Results

### 2.1. Demographics and Clinical Characteristics

A total of 2442 polymyxin prescriptions were tracked over the entire study period (Table 1). Over a period of 4 years post-ASP implementation, 1670 polymyxin prescriptions were evaluated for appropriateness through an ASP committee-driven post-prescriptive audit. A retrospective review of 772 polymyxin prescriptions was done over the pre-implementation period of 1 year.

Polymyxin prescriptions were evaluated for appropriateness, and patients were longitudinally followed up for outcomes across all specialties. During the pre-implementation period (February 2015–February 2016), 772 polymyxin prescriptions were audited. Pneumonia, urinary tract infections (UTIs) and bacteremia were the predominant foci of infection in both medical and surgical specialties necessitating colistin use. During the post-implementation period (February 2016–February 2020), 1670 polymyxin prescriptions were evaluated, and a steady and sustained decrease was recorded in the post-implementation period (Table 1). Polymyxin prescriptions dropped to an average of 417 per year, which amounted to a 45% reduction from the 772 polymyxin prescriptions in the pre-implementation period. The defined daily doses (DDDs) for 1000 patient days depicted a decrease from 34.03 in the pre-ASP period to 13.68 in the last year of the post-implementation period assessed. In terms of the DDD per 100 occupied beds, the DDD value of 2.74 in the pre-implementation phase decreased to 2.06 in the final year of the study period. The decrease in polymyxin prescriptions was reflected across adult medical and surgical departments and pediatric subspecialties. The de-escalation rates of polymyxins which were empirically prescribed in the absence of a culture or when cultures yielded microbes sensitive to second-line antibiotics like BLs (beta-lactam), BLIs (beta-lactamase inhibitors) and tetracyclines showed a general trend of increasing after implementation of the stewardship program (Table S2).

**Figure 1:**
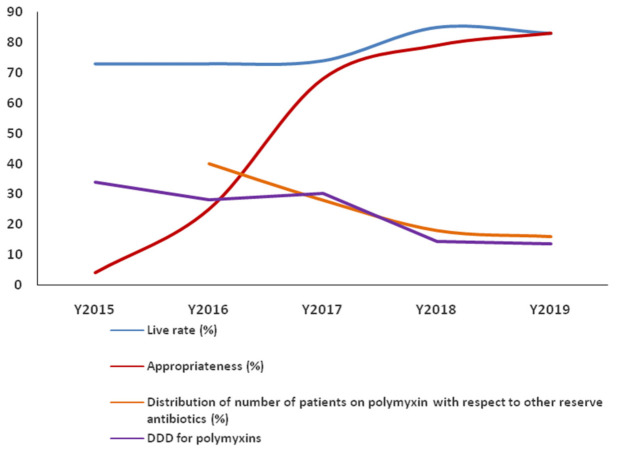
Yearly distribution of survival rates, prescription appropriateness, polymyxin use among reserved drugs and the defined daily dose (DDD) of polymyxins.

The distribution of patients on polymyxins relative to other reserved drugs that exhibited a decreasing trend in general during the 4 years post-implementation of the ASP is depicted in Figure 1. The cost of polymyxin consumption decreased from INR 15.5 crores (USD 2.1 million) in the pre-ASP period to INR 5.7 crores (USD 0.78 million) in the fourth year of the post-implementation period (Figure S1).

### 2.2. Focus of Infection

In the pre-ASP implementation year, pneumonia (n = 306, 40%) was the major focus of infection necessitating the initiation of polymyxins. This was followed by bacteremia (n = 193, 25%) and urinary tract infections (UTIs) (n = 186, 25%). A similar trend was observed for the foci of infection in the years post-ASP implementation (Table 1). Cultures were sent before polymyxin administration in 88% of cases in the pre-ASP implementation period, which rose to 93% after 4 years post-implementation despite a reduction to 68% observed during the second year (2017–2018). Yearly data regarding microbial etiology requiring polymyxin prescriptions revealed a predominance of *Klebsiella pneumoniae* (Table 2), followed by *Acinetobacter baumannii* and *Pseudomonas* species. Colistin susceptibility data revealed preserved sensitivity to colistin in blood isolates among *Klebsiella pneumoniae, Acinetobacter baumannii, Pseudomonas aeruginosa*, *E. coli* and *Enterobacter* species post-ASP implementation (Table S3).

### 2.3. Appropriateness

A prescription was marked as appropriate only if the composite measurement of the 5 Rs criteria was satisfied. Retrospective review of the polymyxin prescriptions in the pre-implementation period revealed an appropriateness of 4% (n = 29), and the major instance of inappropriateness was identified to be the loading dose. An inappropriate loading dose was administered in 93% (n = 725) of polymyxin prescriptions, while 75% (n = 581), 34% (n = 263), 74% (n = 569), 17% (n = 129) and 66% (n = 511) of the maintenance dose, drug, frequency, indication and duration, respectively, were found to be inappropriate (Table 3).

A prospective audit of the prescriptions after ASP implementation revealed an improvement in overall appropriateness to 25% by the end of one year and a further rise to 68%, 79% and 83% at the completion of second, third and fourth years, respectively. The appropriateness of the loading dose improved progressively from 7% to 91% over 4 years. The other components of appropriateness, including the right indication, right drug, right maintenance dose and right frequency, also demonstrated progressive improvement (Table 3). The right indication increased from 69% in the first year of ASP implementation to 97% in the second year and remained almost consistent at 98% and 97% in the third and fourth years, respectively. Similarly, the right drug rates improved from 66% in the first year to 93%, 96% and 95% at the completion of second, third and fourth years, respectively. The right maintenance dose rates demonstrated a progressive increase at an average of 5.25% per year, from 72% in the first year to 93% in the fourth year. The right frequency rates increased from 84% in the first year to 96% in the second year and remained consistent at 98% in the third and fourth years. The right duration improved from 77% in the first year to 90%, 96% and 97% in the second, third and fourth years, respectively.

The combination therapy with optimum synergy advised for polymyxin prescriptions, as per institutional policy, consisted of carbapenems (meropenem, doripenem and ertapenem), tigecycline and fosfomycin. The distribution and overall appropriateness of all co-prescribed reserve antibiotics are depicted in Table S4.

### 2.4. Mortality

The inpatient mortality in patients treated with polymyxin during the pre-implementation year was 27%. There was a sustained year-on-year reduction in mortality over 4 years at a yearly average of 22.25%, translating to a percentage reduction of 17.6%. The yearly survival rates of the patients, along with the appropriateness of prescriptions and DDDs of polymyxins exhibiting a favorable trend, are depicted in Figure 1.

The quarterly overall appropriateness rates of the polymyxin prescriptions were observed to have a significant positive correlation with the survival rates (r = 0.4774, *p* = 0.02). A significant positive correlation was also observed between the quarterly survival rates of patients and the right loading dose (r = 0.5228, *p* = 0.015) and right duration (r = 0.4361, *p* = 0.04) among polymyxin prescriptions over the entire study period.

## 3. Discussion

Epidemiological data on polymyxin use at our hospital revealed an improvement in appropriateness of prescriptions, translating to reduced drug consumption and potential survival benefits in our quasi-experimental study assessing the effectiveness of ASP implementation. A global review of antibiotic consumption revealed that antibiotic utilization increased by 36% over a 10 year period (2000–2010), with the most notable escalation reflected in the carbapenem and polymyxin classes [19]. This trend correlates with the mounting rates of MDR and XDR gram-negative pathogens and mirrors the need to parallelly rationalize the use of polymyxins through strategies like antimicrobial stewardship to prevent the emergence of polymyxin resistance and ensure an optimum clinical cure.

Clinical and microbiological parameters like the focus of infection and the etiological agents remained fairly constant over the years following implementation, suggesting that the survival benefits observed in the study period could potentially be a reflection of the improvement in prescription patterns. The quarterly survival rates were significantly correlated with the rates of the appropriate loading dose and the duration of polymyxin prescriptions. However, no significant correlation was observed between the survival rates and appropriateness rates of the right indication, drug, maintenance dose and frequency despite an overall improvement in their appropriateness. The predominance of *Klebsiella pneumoniae* and *Pseudomonas* species observed in our study cohort mirrored the high prevalence of Gram Negative Bacteria (GNB) reported in the Indian population, warranting the use of reserved drugs like polymyxins in healthcare settings across the country [4,20]. Our study emphasizes the importance and effectiveness of ASP initiatives in improving the indiscriminate prescription patterns of last resort antimicrobials used in the treatment of MDR organisms and potential survival benefits [21].

The goals of antimicrobial stewardship programs include improving prescription patterns for physicians and ensuring antimicrobial resistance surveillance. ASPs are currently sparse and unstructured in India, but they are gaining momentum with the support of the government [3,22]. Hospital policies on polymyxin dosing are vital in ensuring that patients are not subject to either underdosing or overdosing, which can lead to the emergence of resistance or drug toxicities, respectively. However, institutional level policies are scarce due to the paucity of local and regional guidelines on polymyxin use from Indian settings. In addition, data regarding the prescription practices of polymyxins in Indian settings are sparse [23]. The available guidelines highlight the need for a mandatory loading dose on the initiation of polymyxins to ensure that optimal steady state concentrations are attained as early as possible [9,24]. A recent colistin pharmacokinetic study from India highlighted that decreased steady state concentrations correlated with lower clinical efficacy [25]. However, the practice of an appropriate loading dose was extremely low in our setting. The standardization of prescriptions brought about by the implementation of antibiotic stewardship programs was multipronged, with a daily audit of prescriptions with direct feedback to the clinicians regarding practices, the introduction of a color-coded prescription chart specific to antibiotics, the empowerment of nurses in stewardship practices, a regular monthly audit of polymyxin consumption and the stating of drugs needing loading doses in the antibiotic prescription chart for direct reference. Opportunities for further stewardship exist in infections caused by carbapenem-resistant, cephalosporin-susceptible *Pseudomonas aeruginosa*, in which colistin use can be spared [26].

The implementation of the ASP team in our tertiary care academic hospital led to a significant decrease in the number of polymyxin prescriptions over the years, which emphasizes the importance of audit and strategies to optimize antibiotic use in hospital settings. Optimization of the dosage and duration through prospective audit and feedback, culminating in the improvement of appropriateness, and possible survival advantages clearly could represent the use of such strategies in wider settings.

### Limitations

Our study did not include the information on nephrotoxicity, neurotoxicity and other adverse effects reported for polymyxins. Adverse events are monitored by a dedicated pharmacovigilance team at the hospital. With the major focus of the study being polymyxin consumption over the years, we did not incorporate the details of various combination therapies used along with polymyxins. The inclusion of ethnographic and behavioral components that could influence antimicrobial prescription practices were beyond the scope of the current study due to limited resource capacity in LMIC (Low and Middle Income Countries) settings. The association of appropriateness of polymyxin prescriptions with outcome indicators, such as clinical and microbiological cures, were not studied. The repetition of microbiological cultures to confirm negative growth was neither recommended nor financially feasible due to resource constraints.

## 4. Materials and Methods

### 4.1. Study Setting and Population

The quasi-experimental study was undertaken in a 1300 bed tertiary care hospital with 13 intensive care units (ICUs), encompassing a total of 248 ICU beds and having a robust turnover of critically ill patients. An ethical waiver was obtained from the institutional ethics committee of the hospital.

#### Study Population

All inpatient admissions at the hospital who were initiated on PB and PE (colistin) between 15 February 2016 and 15 January 2020 were included.

### 4.2. Program Design and Implementation

A multidisciplinary ASP was established in February 2016 which identified polymyxins as restricted antimicrobial agents from its inception. The strategy of the post-prescriptive audit inculcating the key model (start smart, then focus) for the reserved drug prescriptions in daily stewardship meetings was followed. Recommendations for the appropriate prescription of reserve antibiotics were communicated to the treating clinician whenever the audit showed inappropriate use. The ASP team reviewed and adapted compendium guidelines from the Infectious Diseases Society of America (IDSA), Society for Healthcare Epidemiology of America (SHEA) and the Centers for Disease Control and Prevention (CDC) for the guiding principles of antimicrobial stewardship.

To standardize dosing for polymyxins, the ASP team established and disseminated guidelines for the loading dose and maintenance doses, which were further updated as per the International Consensus Guidelines for the Optimal Use of the Polymyxins [13] (Box 1). Combination therapy was mandated with all polymyxin prescriptions post-ASP implementation. Daily dose optimization based on creatinine clearance was followed. Institution-wise sensitization for the recognition of polymyxin-induced dyselectrolytemia and neurotoxicity was imparted. Due reporting of the polymyxin-induced adverse drug reactions to the pharmacovigilance committee by the primary physician was encouraged.

Box 1. **ASP TARGETED INTERVENTION FOR POLYMYXINS.**

ASP TARGETED INTERVENTION FOR POLYMYXINS

➢ For invasive infections due to multidrug-resistant organisms (MDROs), polymyxin B or colistin should be used in combination with an additional agent.
➢ Patients requiring IV polymyxin therapy for suspected or documented MDR HAP (Hospital-acquired pneumonia) or VAP (Ventilator-associated pneumonia) should receive adjunctive polymyxin aerosol therapy.
➢ Inclusion of the directive in a color-coded antibiotic prescription sheet demanding loading dose administration of colistin and polymyxin.
➢ Empowerment of nurses in stewardship practices. Nurses alert physicians to initiate loading doses for reserved antibiotics like polymyxins, which require a loading dose (LD) to ensure that appropriate loading doses are prescribed.
➢ Follow the Institutional Guidelines for Dosing of Polymyxins (Table S1).
➢ Initiate a first maintenance dose for colistin 12 h after the loading dose, based on the TDM study on Colistin.
➢ Daily dose amendments for creatinine clearance and RRT (Table S1).

### 4.3. Data Collection

A pharmacy consumption report form for polymyxins was provided by the pharmacy to the antimicrobial stewardship team on a daily basis, from which patients initiated on colistin or polymyxin were identified. Data on polymyxin use for 1 year prior to ASP initiation (February 2015–January 2016) was obtained from the hospital information system (HIS). A retrospective chart review was done to evaluate the appropriateness of polymyxin prescriptions during the pre-implementation period of the ASP. The post-implementation period was considered to be from February 2016 to January 2020.

Upon the identification of patients initiated on polymyxin or colistin, a bedside visit was paid by the clinical pharmacist, who was duly trained to capture the clinical data in a specially designed case report form (Appendix A). This involved detailed data collection, including demographics, admitting specialty, diagnosis, details of sending cultures, prescription details including indication for initiating antimicrobials, administration of the right loading dose or maintenance dose, right frequency and duration together with necessary dose adjustments in renal failure, compliance to the 5 Rs model, laboratory parameters and biomarkers and microbiology data, including specimens cultured and organisms isolated along with their susceptibility. Appropriateness was reviewed by the ASP clinical team as per the 5 Rs model (right drug, right indication, right dose, right frequency and right duration) [18] (Table 4). The empiric use of polymyxins was indicated and recommended in clinical conditions, such as suspected gram-negative sepsis with a high risk of Carbapenem-resistant Enterobacteriaceae (CRE) related Hospital acquired infections (HAIs) awaiting microbiological culture results, immunosuppressed or neutropenic patients or patients with breakthrough infections. Definitive use was indicated for culture and sensitive yielding MDR gram-negative bacteria, which is sensitive only to colistin, and for culture-negative cases and HAIs, an institutional antibiogram based on the focus of infection was followed. A prescription was marked as appropriate only if it fulfilled the 5 Rs criteria. Inappropriate use was discussed with providers and often coupled with a stewardship recommendation, which was filed in the patient’s medical chart and discussed with the primary treating team in person or over the phone or email.

The VITEK-2 automated system was used for antimicrobial susceptibility testing. Colistin minimum inhibitory concentrations (MICs) ≤ 2 μg/mL were considered susceptible, and MICs ≥ 4 μg/mL were resistant for all *Enterobacterales*, *Pseudomonas aeruginosa* and *Acinetobacter* species. All-cause mortality during an inpatient stay was recorded as an outcome. The defined daily dose (DDD) per 1000 patient days was used to compare the consumption of polymyxins during the pre-ASP and post-ASP periods and calculated as per CDC National Healthcare Safety Network (NHSN) guidelines [27]. The cost of polymyxin consumption was estimated based on the average cost of the vials of all available brands dispensed across the study period to account for cost fluctuations during the same period.

### 4.4. Statistical Analysis

Descriptive statistics was used to describe the epidemiological data of polymyxin in terms of frequencies. Pearson’s correlation coefficient was employed to investigate correlations among the quarterly survival rates and prescription appropriateness rates. A *p* value of < 0.05 was considered statistically significant. Data were analyzed using SPSS 21.0 for Windows (SPSS Inc., Chicago, IL, USA).

## 5. Conclusions

Our study on the epidemiology of polymyxin use demonstrated favorable effects on the appropriateness of prescriptions and mortality benefits after the successful implementation of antimicrobial stewardship coupled with targeted interventions on polymyxins, including standardized dosing protocols and the optimization of prescriptions as per the 5 Rs model. Multiple stewardship opportunities for improvement exist in LMICs, including the administration of appropriate loading doses, optimizing maintenance doses according to renal function, prioritizing culture-driven prescribing where possible and the appropriate duration of treatment to preserve colistin efficacy for the foreseeable future.

## Figures and Tables

**Table 1:** General characteristics of polymyxin prescriptions over the study period before and after antimicrobial stewardship program (ASP) implementation.

Variables	Pre-ASP Implementation (n)			2016–2017 (n)			2017–2018 (n)			2018–2019 (n)			2019–2020 (n)		
	C	PB	Total	C	PB	Total	C	PB	Total	C	PB	Total	C	PB	Total
	738	34	772	315	95	410	358	128	486	314	62	376	370	28	398
**Age**															
Median (IQR)	55 (30)	50.50 (39)	54 (32)	55 (32)	55 (24)	55 (30)	54 (27)	51.50 (22)	53 (26)	56 (35)	54 (18)	56 (30)	48 (59)	61 (7)	50 (58)
Above 80 years, n (%)	18	0	18 (2%)	9	1	10 (2%)	2	0	2 (0.4%)	10	1	11 (3%)	10	1	11 (3%)
**Sex**															
Male	491	21	512 (67%)	230	65	295 (72%)	227	81	308 (63%)	214	39	253 (67%)	246	21	267 (67%)
**Focus of Infection**															
Pneumonia	289	17	306 (40%)	87	31	118 (29%)	105	41	146 (30%)	137	29	166 (44%)	272	35	307 (77%)
UTI	186	7	193 (25%)	88	11	99 (24%)	103	15	118 (24%)	83	10	93 (25%)	97	3	100 (25%)
Bacteremia	182	8	190 (25%)	92	46	138 (34%)	146	63	209 (43%)	92	20	112 (30%)	127	4	131 (33%)
Skin and soft tissue infection	75	5	80 (10%)	36	3	39 (10%)	34	10	44 (9%)	39	7	46 (12%)	49	2	51 (13%)
Abdominal	7	0	7 (1%)	8	3	11 (3%)	16	7	23 (5%)	16	8	24 (6%)	35	4	39 (10%)
CNS	4	4	8 (1%)	2	1	3 (1%)	12	4	16 (3%)	5	2	7 (2%)	39	0	39 (10%)
Others*	17	0	17 (2%)	0	0	0	52	15	67 (14%)	54	26	80 (21%)	140	19	159 (40%)
**Culture Sent**															
Culture sent prior to antibiotics	649	31	680 (88%)	307	89	396 (96%)	246	90	336 (69%)	292	57	349 (93%)	344	26	370 (93%)
Culture sent after antibiotics	86	2	88 (11%)	8	6	14 (3%)	145	56	201 (41%)	17	4	21 (5%)	27	5	32 (8%)
Culture not sent	3	1	4 (0.5%)	1	0	1 (0.24%)	3	1	4 (1%)	2	1	3 (0.7%)	7	0	7 (2%)
Appropriateness	29	0	29 (4%)	73	30	103 (25%)	241	91	332 (68%)	253	48	301 (79%)	325	22	347 (83%)
**Outcome**															
Mortality	201	10	211 (27%)	91	20	111 (27%)	80	43	123 (25%)	52	14	66 (17%)	69	12	81 (20%)

The percentages depict the proportion of the specified variable among the total of patients prescribed with polymyxins in the particular year given. Others* includes febrile neutropenia, otitis media, infective endocarditis, endophthalmitis, septic arthritis, discitis, sepsis with unknown source and septic shock. **C = colistin, PB = polymyxin B**.

**Table 2:** Year-wise distribution of isolates treated with colistin and polymyxin.

Organism	2016–2017			2017–2018			2018–2019			2019–2020		
	Number of Isolates Treated with Colistin (%)	Number of Isolates Treated with Polymyxin B (%)	Total (336)	Number of Isolates Treated with Colistin (%)	Number of Isolates Treated with Polymyxin B (%)	Total (290)	Number of Isolates Treated with Colistin (%)	Number of Isolates Treated with Polymyxin B (%)	Total (358)	Number of Isolates Treated with Colistin (%)	Number of Isolates Treated with Polymyxin B (%)	Total (455)
***Acinetobacter baumanii***	49 (14%)	13 (4%)	62 (18%)	47 (16%)	7 (2%)	54 (19%)	43 (12%)	8 (2%)	51 (14%)	84 (18%)	8 (2%)	92 (20%)
***E. coli***	23 (7%)	5 (1%)	28 (8%)	30 (10%)	8 (3%)	38 (13%)	27 (7%)	2 (0.5%)	29 (7.5%)	37 (8%)	4 (1%)	41 (9%)
***Enterobacter cloacae***	9 (3%)	2 (0.5%)	11 (3.5%)	5 (2%)	0	5 (2%)	5 (1%)	1 (0.2%)	6 (1.2%)	8 (2%)	1 (0.2%)	9 (2%)
***Klebsiella pneumoniae***	112 (33%)	45 (13%)	157 (46%)	129 (44%)	40 (14%)	169 (58%)	173 (48%)	39 (11%)	212 (59%)	210 (46%)	30 (6%)	240 (53%)
***Pseudomonas aeruginosa***	61 (18%)	17 (5%)	78 (23%)	20 (7%)	4 (1%)	24 (8%)	46 (13%)	14 (4%)	60 (17%)	67 (15%)	6 (1%)	73 (16%)

**Table 3:** Distribution of the 5 Rs of consumption of colistin and polymyxin B.

Variables	Pre-ASP Implementation (n)			2016–2017 (n)			2017–2018 (n)			2018–2019 (n)			2019–2020 (n)		
	C	PB	Total	C	PB	Total	C	PB	Total	C	PB	Total	C	PB	Total
	738	34	772	315	95	410	358	128	486	317	63	380	389	28	417
Right indication	613	30	643 (83%)	225	57	282 (69%)	349	122	471 (97%)	313	61	374 (98%)	377	28	405 (97%)
Right drug	488	21	509 (66%)	215	55	270 (66%)	334	117	451 (93%)	308	59	367 (96%)	375	27	402 (96%)
Loading dose required	642	34	676 (87%)	292	95	387 (94%)	329	121	450 (92%)	291	60	351 (92%)	299	28	327 (78%)
Right loading dose given	46	0	46 (6%)	135	72	207 (53%)	245	103	348 (77%)	262	51	313 (89%)	282	17	299 (91%)
Right maintenance dose given	164	27	191 (25%)	212	84	296 (72%)	307	120	427 (88%)	287	58	345 (91%)	360	27	387 (93%)
Right frequency	174	29	203 (26%)	256	90	346 (84%)	341	125	466 (96%)	311	63	374 (98%)	382	28	410 (98%)
Right duration	247	14	261 (34%)	242	72	314 (77%)	325	114	439 (90%)	305	59	364 (96%)	378	28	406 (97%)

C = colistin; PB = polymyxin B.

**Table 4:** Definition of the 5R criteria used for assessing appropriateness.

Parameter	Definition	Example
Right indication	When the prescribed polymyxin B is the most appropriate selection in terms of site of infection and pathogen	1. Prescribing polymyxin B instead of colistin for a mul-tidrug-resistant *Klebsiella pneumoniae* urinary tract infection is considered inappropriate because polymyxin B does not achieve optimal concentration in the urine. 2. Appropriate escalation to colistin or polymyxin B when the patient clinically does not respond to carbapenems.
Right drug	When colistin or polymyxin B is the narrowest and most effective antibiotic	3. Appropriate tailoring to colistin or polymyxin B based on a culture and sensitivity report. 4. Prescribing colistin alone for bacteremia is considered to be inappropriate (polymyxins should be prescribed with an appropriate syner-gistic agent). 5. Prescribing inhalational colistin alone for pneumonia is considered to be inappropriate.
Right dose	When the loading dose and maintenance dose of the prescribed antimicrobial are appropriate and accurate for the patient’s diagnosis as per standard recommendations	6. Prescribing the appropriate loading dose of colistin irrespective of creatinine clearance is mandatory to achieve an adequate steady state concentration. 7. Prompt dose adjustment based on creatinine clearance and according to body weight for pediatric patients
Right frequency	When the frequency of the prescribed antimicrobial dose is appropriate for the patient’s diagnosis as per standard recommendations	8. Maintenance dose to be administered 12 h after the loading dose and to pursue further frequency based on creatinine clearance.
Right duration	When the prescribed antimicrobial has been administered for the correct duration based on the patient’s diagnosis as per standard recommendations	9. Prescribing colistin for 7–10 days for hospital-acquired pneumonia.

## Data Availability

The data presented in this study are available on request from the corresponding author.

## Supplementary Materials

The following are available online at https://www.mdpi.com/article/10.3390/antibiotics10050470/s1. Table S1: Institutional dosing guidelines for Colistin and Polymyxin B, Table S2: Details of empiric prescription patterns related to polymyxins involving de-escalation and continuation of the polymyxins, Table S3: Percentage susceptibility of Colistin, Table S4: Distribution and appropriateness of co-prescribed reserved antimicrobials along with polymyxins and Figure S1: Cost of polymyxin consumption

## Appendix A

	**Amrita Institute of Medical Sciences and Research Centre** (an ISO 9001/14001/18001/NABH/NABL/NAAC certified hospital) **ANTIBIOTIC STEWARDSHIP COMMITTEE**						
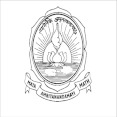
**Data Collection Form**							
1. Name of the patient							
2. MRD No:							
3. Date of Admission				4. Date of Review			
5. Age in years				6. Sex: Male/Female			
7. Location							
8. Admitting Doctor							
9. Admission Diagnosis							
10. Suspected focus of infection							
(a) Pneumonia							
(b) UTI							
(c) CNS							
(d) Skin and Soft Tissue							
(e) Abdominal							
(f) Bacteremia							
(g) Catheter/Lines/Stents							
(h) Other:							
11. Cultures							
(A) Culture sent- Yes/No							
(B) Date and time of culture sent:							
(C) Sample sent for culture							
a. Blood							
b. Urine							
c. Stool							
d. Sputum							
e. Mini Bal							
f. CSF							
g. Ascitic fluid							
h. Pleural fluid							
i. Tissue							
j. Pus							
(D) Provisional report of culture—after 48 h of sending (To include culture and sensitivity report if available)							
12. S. Creatinine (mg/dL)							
13. Antibiotics used							
Antibiotic		Dose	Route	Frequency	Date of initiation	Loading dose	Infusion
						
						
						
						
						
14. Clinical Signs correlating with Antibiotic initiation (prior 48 h)							
Temp (°F)-							
BP (mmHg)-							
RR (per minute)-							
O2 saturation (%)-							
WBC (K/uL)-							
CRP (mg/L)-							
Procalcitonin (ng/mL)-							
Lactate (mmol/L)-							
At 48 h							
Antibiotics changed							
							

## References

[1] Prestinaci F., Pezzotti P., Pantosti A. (2015). Antimicrobial resistance: A global multifaceted phenomenon. Pathog. Glob. Health.

[2] Goff D.A., Kaye K.S. (2014). Minocycline: An Old Drug for a New Bug: Multidrug-Resistant Acinetobacter baumannii. Clin. Infect. Dis..

[3] Kakkar M., Walia K., Vong S., Chatterjee P., Sharma A. (2017). Antibiotic resistance and its containment in India. BMJ.

[4] Nagvekar V., Sawant S., Amey S. (2020). Prevalence of multidrug-resistant Gram-negative bacteria cases at admission in a multispeciality hospital. J. Glob. Antimicrob. Resist..

[5] Aslam A., Gajdács M., Zin C.S., Ab Rahman N.S., Ahmed S.I., Zafar M.Z., Jamshed S. (2020). Evidence of the Practice of Self-Medication with Antibiotics among the Lay Public in Low- and Middle-Income Countries: A Scoping Review. Antibiotics.

[6] Van Duin D., Paterson D.L. (2016). Multidrug-Resistant Bacteria in the Community. Infect. Dis. Clin. N. Am..

[7] Garg S.K., Singh O., Juneja D., Tyagi N., Khurana A.S., Qamra A., Motlekar S., Barkate H. (2017). Resurgence of Polymyxin B for MDR/XDR Gram-Negative Infections: An Overview of Current Evidence. Crit. Care Res. Pract..

[8] Budd E., Cramp E., Sharland M., Hand K., Howard P., Wilson P., Wilcox M., Muller-Pebody B., Hopkins S. (2019). Adaptation of the WHO Essential Medicines List for national antibiotic stewardship policy in England: Being AWaRe. J. Antimicrob. Chemother..

[9] Kadri S.S., Adjemian J., Lai Y.L., Spaulding A.B., Ricotta E., Prevots D.R., Palmore T.N., Rhee C., Klompas M., Dekker J.P. (2018). National Institutes of Health Antimicrobial Resistance Outcomes Research Initiative (NIH–ARORI). Difficult-to-Treat Resistance in Gram-negative Bacteremia at 173 US Hospitals: Retrospective Cohort Analysis of Prevalence, Predictors, and Outcome of Resistance to All First-line Agents. Clin. Infect. Dis..

[10] (2017). Antibiotic Resistance: ICMR Advises Hospitals to Avoid Three Antibiotics. Med. Dialogues.

[11] Walia K., Ohri V., Madhumathi J., Ramasubramanian V. (2019). Policy document on antimicrobial stewardship practices in India. Indian J. Med. Res..

[12] Walia K., Madhumathi J., Veeraraghavan B., Chakrabarti A., Kapil A., Ray P., Singh H., Sistla S., Ohri V. (2019). Establishing Antimicrobial Resistance Surveillance & Research Network in India: Journey so far. Indian J. Med. Res..

[13] Tsuji B.T., Pogue J.M., Zavascki A.P., Paul M., Daikos G.L., Forrest A., Giacobbe D.R., Viscoli C., Giamarellou H., Karaiskos I. (2019). International Consensus Guidelines for the Optimal Use of the Polymyxins: Endorsed by the American College of Clinical Pharmacy (ACCP), European Society of Clinical Microbiology and Infectious Diseases (ESCMID), Infectious Diseases Society of America (IDSA), International Society for Anti-infective Pharmacology (ISAP), Society of Critical Care Medicine (SCCM), and Society of Infectious Diseases Pharmacists (SIDP). Pharmacother. J. Hum. Pharmacol. Drug Ther..

[14] Ismail B., Shafei M.N., Harun A., Ali S., Omar M., Deris Z.Z. (2018). Predictors of polymyxin B treatment failure in Gram-negative healthcare-associated infections among critically ill patients. J. Microbiol. Immunol. Infect..

[15] Laxminarayan R., Matsoso P., Pant S., Brower C., Røttingen J.-A., Klugman K., Davies S. (2016). Access to effective antimicrobials: A worldwide challenge. Lancet.

[16] Dixit A., Kumar N., Kumar S., Trigun V. (2019). Antimicrobial Resistance: Progress in the Decade since Emergence of New Delhi Metallo-β-Lactamase in India. Indian J. Community Med..

[17] Ranjalkar J., Chandy S.J. (2019). India’s National Action Plan for antimicrobial resistance–An overview of the context, status, and way ahead. J. Fam. Med. Prim. Care.

[18] Singh S., Menon V.P., Mohamed Z.U., Kumar V.A., Nampoothiri V., Sudhir S., Moni M., Dipu T.S., Dutt A., Edathadathil F. (2019). Implementation and Impact of an Antimicrobial Stewardship Program at a Tertiary Care Center in South India. Open Forum Infect. Dis..

[19] Van Boeckel T.P., Gandra S., Ashok A., Caudron Q., Grenfell B.T., A Levin S., Laxminarayan R. (2014). Global antibiotic consumption 2000 to 2010: An analysis of national pharmaceutical sales data. Lancet Infect. Dis..

[20] Laxminarayan R., Chaudhury R.R. (2016). Antibiotic Resistance in India: Drivers and Opportunities for Action. PLoS Med..

[21] Erika M.C., D’Agata E.M., Tran D., Bautista J., Shemin D., Grima D. (2018). Clinical and Economic Benefits of Antimicrobial Stewardship Programs in Hemodialysis Facilities. A Decision Analytic Model. CJASN.

[22] Singh S., Charani E., Devi S., Sharma A., Edathadathil F., Kumar A., Warrier A., Shareek P.S., Jaykrishnan A.V., Ellangovan K. (2021). A road-map for addressing antimicrobial resistance in low- and middle-income countries: Lessons learnt from the public private participation and co-designed antimicrobial stewardship programme in the State of Kerala, India. Antimicrob. Resist. Infect. Control..

[23] Charani E., Castro-Sanchez E., Sevdalis N., Kyratsis Y., Drumright L., Shah N., Holmes A. (2013). Understanding the Determinants of Antimicrobial Prescribing Within Hospitals: The Role of “Prescribing Etiquette”. Clin. Infect. Dis..

[24] Avedissian S.N., Liu J., Rhodes N.J., Lee A., Pais G.M., Hauser A.R., Scheetz M.H. (2019). A Review of the Clinical Pharmacokinetics of Polymyxin B. Antibiotics.

[25] Moni M., Sudhir A.S., Dipu T.S., Mohamed Z., Prabhu B.P., Edathadathil F., Balachandran S., Singh S.K., Prasanna P., Menon V.P. (2020). Clinical efficacy and pharmacokinetics of colistimethate sodium and colistin in critically ill patients in an Indian hospital with high endemic rates of multidrug-resistant Gram-negative bacterial infections: A prospective observational study. Int. J. Infect. Dis..

[26] Behzadi P., Baráth Z., Gajdács M. (2021). It’s Not Easy Being Green: A Narrative Review on the Microbiology, Virulence and Therapeutic Prospects of Multidrug-Resistant Pseudomonas aeruginosa. Antibiotics.

[27] CDC Patient Safety Component Protocol. The National Healthcare Safety Network (NHSN) Manual. https://www.dhcs.ca.gov/provgovpart/initiatives/nqi/Documents/NHSNManPSPCurr.pdf.

