# Supplementary material for: Antimicrobial Stewardship and Its Impact on the Changing Epidemiology of Polymyxin Use in a South Indian Healthcare Setting
Source: Antibiotics (Basel). 2021 Apr 21;10(5):470. doi: 10.3390/antibiotics10050470 (PMC8142974; doi:10.3390/antibiotics10050470)
Supplement: Supplementary file 1 [file antibiotics-10-00470-s001.zip › antibiotics-1157377-supplementary.pdf]

antibiotics

MDPI

Table S1. Amrita antimicrobial stewardship dosing guideline of polymyxin B 2018.

Weight of the Patient (Kg)	Loading Dose	Maintenance Dose
50–69 Kg	15 Lakh units IV stat	10 Lakh units IV q12 hr
70–89 Kg	20 Lakh units IV stat	10 Lakh units IV q12 hr
<50 Kg and > 90 Kg (Adjusted body weight should be used to calculate the dose)	3mg/Kg/dose	1.5mg/Kg q12 hr

Loading dose is required for all patients to achieve steady state concentration rapidly. Maintenance dose should be administered 12 hours after the administration of loading dose. Since polymyxin B is eliminated through non- renal pathways and the serum concentration is not significantly affected by renal function, dosage adjustment in altered renal function is not recommended. *Adjusted body weight (Kg) = Ideal body weight + 0.4(Actual body weight – Ideal body weight).

References

1. Kassamali Z, Jain R, Danziger L. An Update on the arsenal for multidrug-resistant Acinetobacter infections: Polymyxin antibiotics. *International Journal of Infectious Diseases*. 2015; 30:125-132.
2. Sandri A, Landersdorfer C, Jacob J, Boniatti M, Dalarosa M, Falci D, Behle T, Bordinhão R, Wang J, Forrest A, Nation R, Li J, Zavascki A. Population Pharmacokinetics of Intravenous Polymyxin B in Critically Ill Patients: Implications for Selection of Dosage Regimens. *Clinical Infectious Diseases*. 2013; 57(4):524-531.
3. Miglis C, Rhodes N, Avedissian S, Kubin C, Yin M, Nelson B, Pai M, Scheetz M. Population Pharmacokinetics of Polymyxin B in Acutely Ill Adult Patients. *Antimicrob Agents Chemother*. 2018; 62(3):e01475-17.
4. Kubin C, Nelson B, Miglis C, Scheetz M, Rhodes N, Avedissian S, Cremers S, Yin M. Population Pharmacokinetics of Intravenous Polymyxin B from Clinical Samples. *Antimicrob Agents Chemother*. 2018; 62(3):e01493-17.
5. Thamlikitkul V, Dubrovskaya Y, Manchandani P, Ngamprasertchai T, Boonyasiri A, Babic J, Tam V. Dosing and Pharmacokinetics of Polymyxin B in Patients with Renal Insufficiency. *Antimicrob Agents Chemother*. 2016; 61(1):e01337-16.

Recommendation discussed with: Dr.Vidya.P.Menon, Dr.Syam Sunder, Dr.Zubair Umer Mohammed, Dr.Sabarish.B.Nair, Dr.Merlin Moni, Dr.Dipu T.S, Infectious disease clinical pharmacists

Amrita Antimicrobial Stewardship Dosing Guideline of Colistin 2019

Loading dose – 9MU in 100ml NS over 30minutes to 1 hour irrespective of creatinine clearance to achieve steady state plasma concentration early.

Maintenance dose needs to be initiated 12 hours after the administration of loading dose based on the following categories of creatinine clearance[1,2]

Creatinine Clearance (ml/min)	Maintenance Dose of Colistin
≥80mL/min	**9MU/day** i.e 3MU IV q8 hr
50 to <80 mL/min	**8MU/day** i.e 4MU IV q12 hr
30 to <50 mL/min	**6MU/day** i.e 3MU IV q12 hr
<30mL/min	**4MU/day** i.e 2MU IV q12 hr
Intermittent HD and Maintenance HD	**Non-dialysis days** – 2 MU IV q12hr<br>**Dialysis days-** 2MU IV q12hr and 1MU supplemental dose after HD<br>Conduct IHD session as late as possible within the dose interval (HD to be completed before next dose) to minimize the amount of CMS and formed colistin lost to the extracorporeal system
SLED (Sustained Low Efficiency Dialysis) Approx 8-10 hrs	2 MU IV q12hr (i.e. 4MU/day) and supplemental dose of 0.4MU (10% of 130 mg CBA) per each hour of SLED to be given with the baseline dose. (i.e for a 10hr SLED, 8MU / day (4MU baseline dose and 4MU supplemental dose) can be safely and effectively administered as 4MU IV q12 hr
Continous Renal Replacement Therapy	13 MU per day (440mg CBA) which can be administered as 6.5 MU IV q12 hr

For obese patients, Ideal body weight should be used to determine the colistin dose.

References

1. Nation RL, Garonzik SM, Li J, et al. Updated US and European Dose Recommendations for Intravenous Colistin: How Do They Perform? *Clin Infect Dis*. 2016;62(5):552-558. doi:10.1093/cid/civ964
2. Nation RL, Garonzik SM, Thamlikitkul V, et al. Dosing guidance for intravenous colistin in critically-ill patients. *Clin Infect Dis*. December 2016:ciw839. doi:10.1093/cid/ciw839
3. Tsuji BT, Forrest A, Giacobbe DR, Viscoli C, Giamarellou H, Karaiskos I. S PECIAL A RTICLE International Consensus Guidelines for the Optimal Use of the Polymyxins : doi:10.1002/phar.2209

Table S2. Details of empiric prescription patterns related to polymyxins involving de-escalation and continuation of the polymyxins.

**Change in Empirical Prescription Pattern**	**2016–2017**	**2017–2018**	**2018–2019**	**2019–2020**
	N = 121	N = 52	N = 31	N = 174
Empiric polymyxins continued	86(71%)	34(65%)	16(52%)	118(68%)
Empiric polymyxins de-escalated	34 (28%)	19(36%)	15(48%)	56(32%)

Table S3. Percentage Susceptibility of Colistin.

**Percentage Susceptibility of Colistin***	***Klebsiella pneumoniae***			***E.coli***			***Pseudomonas aeruginosa***			***Acinetobacter baumannii***			***Enterobacter sp***		
	2017	2018	2019	2017	2018	2019	2017	2018	2019	2017	2018	2019	2017	2018	2019
	89%	90%	90%	99%	95%	100%	100%	98%	99%	98%	97%	99%	87%	85%	91%

*Blood isolates.

Table S4. Distribution and appropriateness of co-prescribed reserved antimicrobials along with polymyxins.

**Prescription Characteristics**	**2016–2017**	**2017–2018**	**2018–2019**	**2019–2020**
Number of prescriptions				
CARBAPENEMS	587	1352	1582	2011
TIGECYCLINE	96	105	71	166
FOSFOMYCIN	NA	25	29	17
Appropriateness of co-prescribed reserve antibiotics				
CARBAPENEMS	59%	63%	84%	87%
TIGECYCLINE	44%	44%	89%	92%
FOSFOMYCIN	100%	57%	95%	87%

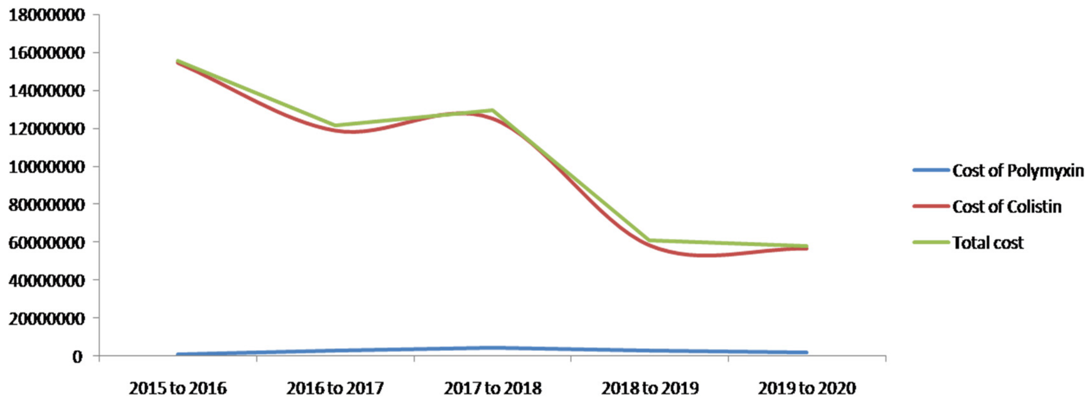

Figure S1. Cost of polymyxin consumption.
